# The Impacts of the Multispecies Approach to Caffeine on Marine Invertebrates

**DOI:** 10.3390/toxics12010029

**Published:** 2023-12-30

**Authors:** Clement Baracchini, Lucie Messager, Philippe Stocker, Vincent Leignel

**Affiliations:** Laboratoire BIOSSE, Le Mans Université, Venue Olivier Messiaen, 72085 Le Mans, France; clement.baracchini@gmail.com (C.B.); philippe.stocker42@gmail.com (P.S.)

**Keywords:** caffeine, marine invertebrates, neurotoxicity, oxidative stress

## Abstract

Caffeine is one of the most consumed substances by humans through foodstuffs (coffee, tea, drugs, etc.). Its human consumption releases a high quantity of caffeine into the hydrological network. Thus, caffeine is now considered an emergent pollutant sometimes found at high concentrations in oceans and seas. Surprisingly, little research has been conducted on the molecular responses induced by caffeine in marine organisms. We studied, in laboratory conditions, six phylogenetically distant species that perform distinct ecological functions (*Actinia equina* and *Aulactinia verrucosa* (cnidarians, predator), *Littorina littorea* (gastropod, grazer), *Magallana gigas* (bivalve, filter-feeder), and *Carcinus maenas* and *Pachygrapsus marmoratus* (crabs, predator and scavenger)) subjected to caffeine exposure. The antioxidant responses (catalase, CAT; glutathione peroxidase, GPx; superoxide dismutase, SOD), lipid peroxidation (MDA), and the acetylcholinesterase (AChE) activity were estimated when the organisms were exposed to environmental caffeine concentrations (5 μg/L (low), 10 μg/L (high)) over 14 days. Differential levels of responses and caffeine effects were noted in the marine invertebrates, probably in relation to their capacity to metabolization the pollutant. Surprisingly, the filter feeder (*M. gigas*, oyster) did not show enzymatic responses or lipid peroxidation for the two caffeine concentrations tested. The marine gastropod (grazer) appeared to be more impacted by caffeine, with an increase in activities for all antioxidative enzymes (CAT, GPx, SOD). In parallel, the two cnidarians and two crabs were less affected by the caffeine contaminations. However, caffeine was revealed as a neurotoxic agent to all species studied, inducing high inhibition of AChE activity. This study provides new insights into the sublethal impacts of caffeine at environmentally relevant concentrations in marine invertebrates.

## 1. Introduction

Caffeine is one of the most commonly consumed substances in humans [1,2]. Caffeine is metabolised in the liver and excreted by the kidney (5% of the native form is excreted) [3]. Caffeine (3,7-dihydro-1,3,7-trimethyl-1h-purine-2,6-dione) is mainly metabolised in 1.7 dimethyxanthine (paraxanthine) [4]. This molecule and its metabolites are released into the environment via urine and production processes. The half-life of caffeine in water ranges from 100 to 234 days [5]. Caffeine is now an emerging contaminant with several hot spots of contamination worldwide in oceans and seas [5]. In marine environments, caffeine concentrations were recently estimated in Europe to be 4.9–677 ng/L in the North Sea (Germany, Holland, Norway, Sweden), 4–804 ng/L in the Atlantic Ocean (France, Portugal), 4.5–3390 ng/L in the Mediterranean Sea (Greece, Turkey), and 8.2–1110 ng/L in the Adriatic Sea (Italy) [6]. High caffeine concentrations were detected along the coasts of the USA (5860 ng/L), the Red Sea (7708 ng/L), Japan (8230 ng/L), Lebanon (10,200 ng/L), Australia (11,000 ng/L) and Brazil (19,300 ng/L) [6]. Thus, caffeine is recognised as a ubiquitous contaminant in aquatic systems [7], and it is now considered a high-priority environmentally hazardous pharmaceutically active compound pollutant in aquatic ecosystems. In this case, it is crucial to evaluate its toxicity in marine organisms [8].

In marine invertebrates, several studies have revealed oxidative stress in polychaetes (*Arenicola marina*, *Diopatra neapolitana*, *Hediste diversicolor*) and an increase in the enzymatic activities involved in biotransformation (GST, and CYP3A4) when animals are exposed for 28 days to a range of concentrations from 0.5 to 8 µg/L caffeine [8]. In marine bivalves (*Mytilus californianus*, *Ruditapes philippinarum*), an increase in lipid peroxidation was also noted over 28 days using caffeine concentrations ranging from 0.01 to 18 µg/L [9]. The same observation was made in an amphipod (*Ampelisca brevicornis*) [8]. In parallel, a decrease in the embryo-larval development of sea urchin (*Paracentrotus lividus*) also emerged at caffeine concentrations from 0.01 to 15 µg/L [10]. Surprisingly, no study has been developed to estimate the effects of caffeine on other marine invertebrates performing distinct ecological functions as grazers or scavengers, and to evaluate the effects of this compound at environmental concentrations.

The aim objective of this study was to determine the precocious effects of caffeine at concentrations detected in oceans and seas (5 μg/L (low) and 10 μg/L (high)) on distinct taxa involved in different ecological niches. We investigated *Actinia equina* and *Aulactinia verrucosa* (Anthozoa, cnidarians, carnivore predator), *Littorina littorea* (Gastropoda, grazer), *Magallana gigas* (Bivalvia, filter-feeder), *Carcinus maenas* and *Pachygrapsus marmoratus* (Malacostraca, crabs, omnivore predator and scavenger)). The activities of antioxidant enzymes (catalase, GPx, SOD) and acetylcholinesterase (AChE) and lipid peroxidation were measured for 14 days. We hypothesised that caffeine exposure can induce stress (neurotoxicity and sublethal responses) in the different model species studied.

## 2. Materials and Methods

### 2.1. Biological Materials

#### 2.1.1. Bivalve

**Figure 1:**
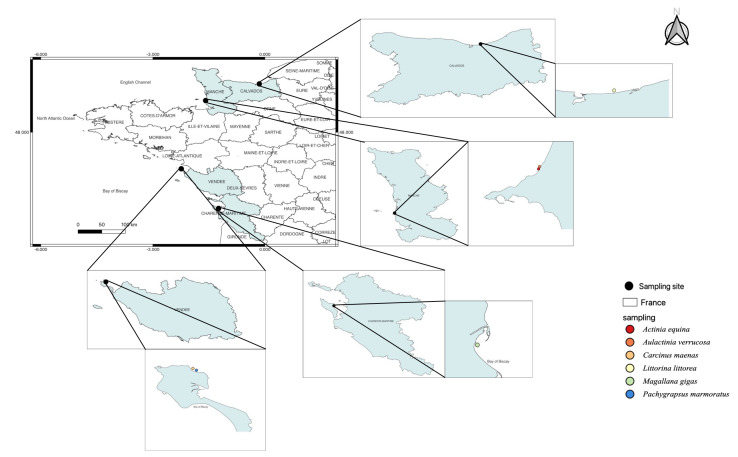
Species sampling map to distinguish marine invertebrates used in this study.

*Magallana gigas*, a Pacific oyster, is the third most cultivated bivalve in the world and has a high economic value in France. This species is used as a model species in ecotoxicology to assess anthropogenic impacts [11]. Thus, this species is commonly used in biomonitoring programs. The 18 oysters used in this study originated from Oleron (Latitude: 45°95′87″ N, Longitude: −1°24′13″ W, Figure 1).

#### 2.1.2. Gastropod

*Littorina littorea* (periwinkle) is abundant in the intertidal zone from Southern Portugal (Atlantic Ocean) to the White Sea (Russia). On the North American coast, *L. littorea* is considered an invasive species. This common gastropod is a good sentinel species [12]. A total of 30 gastropods were found in the Seine Bay (Latitude: 49°28′93″ N, Longitude: −0°17′88″ W, Figure 1).

#### 2.1.3. Cnidarians (Sea Anemone)

*Actinia equina* and *Aulactinia verrucosa* (Sea anemone, cnidaria) are benthic organisms with low mobility common in the Mediterranean Sea and the Atlantic Ocean. Several cnidarians were previously used as a putative sentinel for monitoring environmental pollution [13], to estimate oxidative stress [14,15]. *Actinia equina* is one of the most common species along the French Atlantic coast. A total of 33 *A. equina* and 12 *A. verrucosa* individuals were collected from Donville sur mer (Normandy, Latitude: 48°85′26″ N, Longitude: −1°58′38″ W, Figure 1).

#### 2.1.4. Crustaceans (crabs)

*Carcinus maenas* and *Pachygrapsus marmoratus* (Crustacean, Decapoda) are key components of estuarine and coastal trophic networks [16,17]. Therefore, these species are recognised as a sentinel to analyse the bioaccumulation of inorganic and organic pollutants [18,19,20,21,22]. *Carcinus maenas* is a native species from the East Atlantic Ocean, but also an invader species in the West Atlantic and Pacific Oceans [19]. *Pachygrapsus marmoratus* has also a large geographical distribution (Mediterranean Sea, Black Sea, and Northeastern Atlantic Ocean). Their biology, ecology, and genetics are well documented and contribute to their suitability as research subjects in this field [16,19]. This species is recognised as a sentinel in ecotoxicology [22]. We sampled 18 *C. maenas* and 15 *P. marmoratus* on the foreshore of Noirmoutier en l’île (Latitude: 47°02′50″ N, Longitude: −2°24′92″ W, Figure 1).

**Figure 2:**
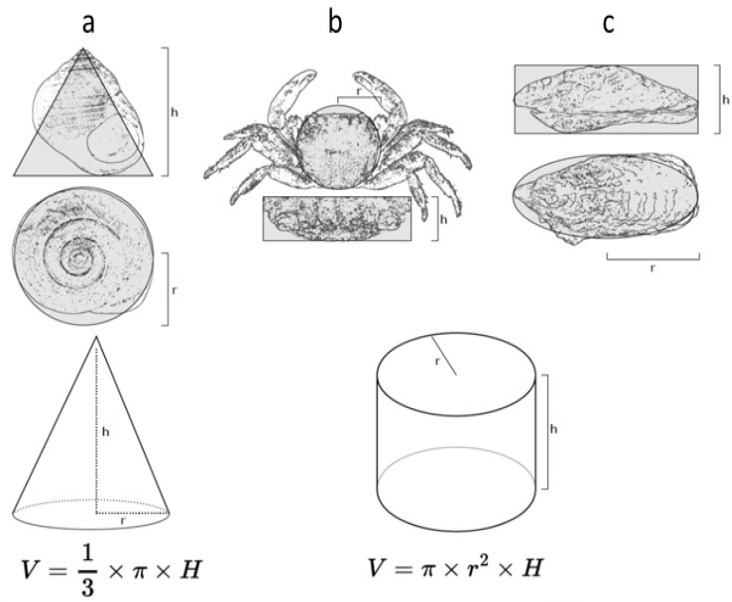
Diagrams showing the morphometric measurements used to calculate the volumes of individuals of the distinct species: (**a**) *Littorina littorea*, (**b**) *Pachygrapsus marmoratus*, and (**c**) *Magallana gigas.* The shape of the gastropod has been simplified to a cone, and that of the crab and oyster to a cylinder to estimate the volumes.

To ensure the homogeneity of distinct groups (control, 5 µg/L, 10 µg/L) for the periwinkles, crabs and oysters, the individuals were measured to estimate a volume. The calculation of the volume used the formula indicated in Figure 2. For the sea anemones, we weighed the individuals.

### 2.2. Acclimatization and Contaminations in the Laboratory

All individuals were acclimatised in the laboratory for 10 days, in aquariums (10 L, Instant Ocean artificial water 33 g/L, lighting: 15 L/9 N, 20 ± 1 °C, pH 7.4–7.5) equipped with bubblers. After acclimatization, three experimental groups were defined: control, 5 µg/L, and 10 µg/L of caffeine.

Each experimental group was composed of 10 *L. littorea* (gastropod), 6 *M. gigas* (bivalve), 6 *C. maenas* (crab), 5 *P. marmoratus* (crab), 11 *A. equina* (Sea anemone), or 4 *A. verrucosa* (Sea anemone).

The caffeine (C_8_H_10_N_4_O_2_, CAS number: 58-08-02, 194.19 g/mol) used was purchased from Merck Millipore. Contaminations were carried out for 14 days. The aquarium water changed once a week. The animals were not fed for the duration of the experiment. The mortality of all individuals was assessed every 24 h by stimulating the foot (*L. Littorina*), the gills (*M. gigas*), or the tentacles (*A. equina*, *A. verrucosa*) of the individuals with fine forceps, or by observation of the general activity (*C. maenas*, *P. marmoratus*). At the end of the 14 days, all individuals were frozen at −80 °C for the biomarker analyses. The molecular responses were estimated separately in the gills and the digestive tract in bivalves and crabs, because these tissues are major organs of xenobiotic and oxyradical-generating biotransformation enzymes [23].

### 2.3. Protein Extraction

One gram of tissues per individual was thawed on ice and ground using mortar and potter in 2 mL of tris-buffered saline (TBS) (pH: 7.4). The homogenate was centrifuged twice at 10,000× *g* at 4 °C for 20 min. The supernatant was kept at −20 °C for further short-term measurements of enzymatic activities. Proteins were quantified as mg/mL according to the Lowry method [24] using a bovine serum albumin (BSA) calibration curve. Absorbance was measured at 490 nm. The R^2^ of the curve was 0.99.

### 2.4. Antioxidative Enzyme Activities

Super oxide dismutase (SOD) activity was estimated using the colorimetric method developed by [25] using a riboflavin/methionine complex generating superoxide anions and nitro-blue tetrazolium (NBT). The coloration was measured at 560 nm and the enzymatic activity was expressed as units (U)/mg protein of SOD activity.

Catalase (CAT) activity was evaluated following the consumption of H_2_O_2_, inducing a decrease in absorbance at 240 nm for 5 min. The reaction took place in phosphate buffer (100 mM) at pH 7.0 at 25 °C with H_2_O_2_ (500 mM) and total protein (1–1.5 mg protein/mL) according to [26]. The CAT activity was expressed by µmoles/min/mg protein.

GPx activity depended on the reaction between the GSH and H_2_O_2_. The enzymatic activity was measured at 420 nm [27]. The protein supernatant was added to 1 mM GSH (2v), and H_2_O_2_ (1.3 mM) was added to initiate the reaction. After 10 min, 1% of the trichloroacetic acid (TCA) was mixed to stop the reaction. After centrifugation, the supernatant was added to Na_2_HPO_4_ (320 mM) and Ellman’s reagent [5,5-Dithiobis(2-nitrobenzoic acid)] (DNTB) (1 mM). The GPx activity was expressed in µmoles of oxidised GSH/min/mg protein.

### 2.5. Lipid Peroxidation

The malondialdehyde (MDA) level was estimated according to the protocol of [28]. Thus, 0.5 mL of protein supernatant was added to TBS and TCA (20%)-BHT (0.01%) and incubated at 100 °C for 30 min. Centrifugation at 10,000× *g* for 10 min allowed us to obtain a supernatant (0.5 mL), which was added to 1 mL of thiobarbituric acid TBA) solution (0.67%), 40 µL of HCl (0.6 M), and incubated for 15 min at 90 °C. The absorbance of the TBA–MDA complex was measured at 532 nm. The MDA concentration was calculated using its molar extinction coefficient (ε= 155 mM/cm). MDA was expressed in nmoles/mg protein.

### 2.6. Acetylcholinesterase Activity

Acetylcholinesterase activity was determined using the [29] method. A phosphate buffer was added volume/volume to a protein mass of 0.6 mg. The reaction mixture contained 0.1 M Tris HCL buffer, 50 µL of DNTB (1 mM). The wavelengths were incubated for 5 min à room temperature. Some 50 µL AchE (45 mM) was added, and each minute (1, 2, 3 and 4 min), the optical density was evaluated at 415 nm. AChE activity was expressed in nmoles of hydrolysed acetylthiocholine per minute per mg of protein (nmoles/min/mg protein).

### 2.7. Statistical Analyses

The study employed a comprehensive multi-biomarker strategy. A dataset was meticulously compiled to focus on these specific species and was categorised by variables such as batch, species, tissue types, and temporal intervals. Statistical software data analysis was conducted using R software (version 4.0.5), taking advantage of packages including Tidyverse for data manipulation. Spatial data regarding the sample locations were mapped using QGIS software version 3.0.2. For data treatment, before any statistical inference, tests for normality and homogeneity of variance were performed using the Shapiro–Wilk and Bartlett’s tests, respectively. Data were presented as mean ± standard deviation within each stratified group (i.e., batch, species, tissue types, and temporal intervals). The normalization mean and standard deviation values of each biomarker were normalised between 0 and 1 within species groups to allow for inter-species comparisons. For statistical tests, we used Welch’s *t*-test to assess the significance of differences between concentrations and control groups for each biomarker. This method was chosen for its robustness against unequal variances and sample sizes, commonly encountered in ecotoxicological studies. To find significance levels, differences were considered statistically significant when the *p*-value was less than 0.05. To facilitate interpretation, *p*-values were converted into asterisks for significance representation on plots. These annotations were positioned above the standard deviation bars. For data presentation, visual plots were constructed, with asterisks placed just above the top of the standard deviation bars to indicate the level of significance.

## 3. Results

### 3.1. Standardisation of Samples Used in Experiments

The estimation of the volume (*Carcinus maenas*, *Littorina littorea*, *Magallana gigas*, and *Pachygrapsus marmoratus*) or the weight *(Actinia equina*, and *A. verrusoca*) allowed us to standardise the experimental groups (Table 1 and Table 2).

### 3.2. Caffeine Effects on Marine Invertebrates

Antioxidative (catalase, GPx, SOD), neurotoxic (AChE) biomarkers, and lipid peroxidation (MDA) showed different responses according to the species studied and the caffeine concentration used.

**Figure 3:**
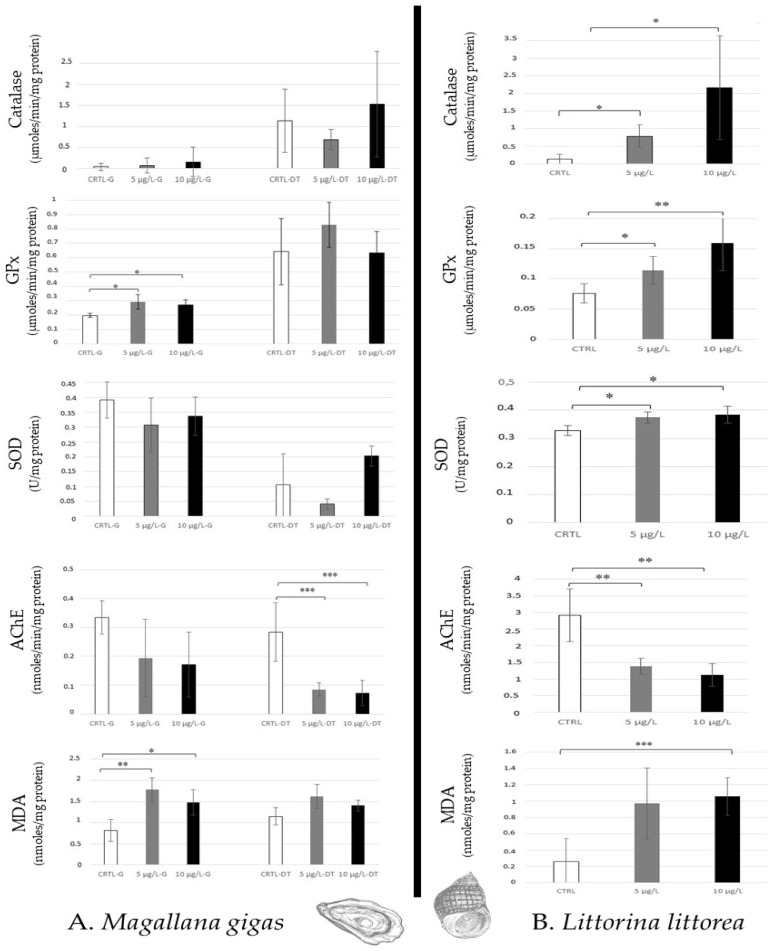
Antioxidative enzymatic responses (Catalase, GPx, SOD), acetylcholinesterase activity, and lipid peroxidation in molluscs (*Magallana gigas*, (**A**); *Littorina littorea*, (**B**)) after 14 days of caffeine contamination (control, CTRL; 5 µg/L; 10 µg/L). Distinct symbols indicate *p*-values: * *p* < 0.05, ** *p* < 0.01, *** *p* < 0.005.

Even if *Mallagana. gigas* filters a high quantity of seawater per hour (from 2 to 5 L/h), which favours the absorption of chemical compounds in tissues, only lipid peroxidation and an increase in GPx activity (no catalase and SOD) were noted in gills for the two environmental caffeine concentrations tested (5 µg/L, 10 µg/L). A high AChE decrease was observed in the digestive tract, whereas no lipid peroxidation and antioxidative responses were detected (Figure 3A).

In opposition, *Littorina littorea*, gastropod (grazer) showed an increase in all antioxidative responses (Catalase, GPx, SOD) and a high AChE decrease for the two caffeine concentrations. Lipid peroxidation was noted at 10 µg/L caffeine (Figure 3B).

**Figure 4:**
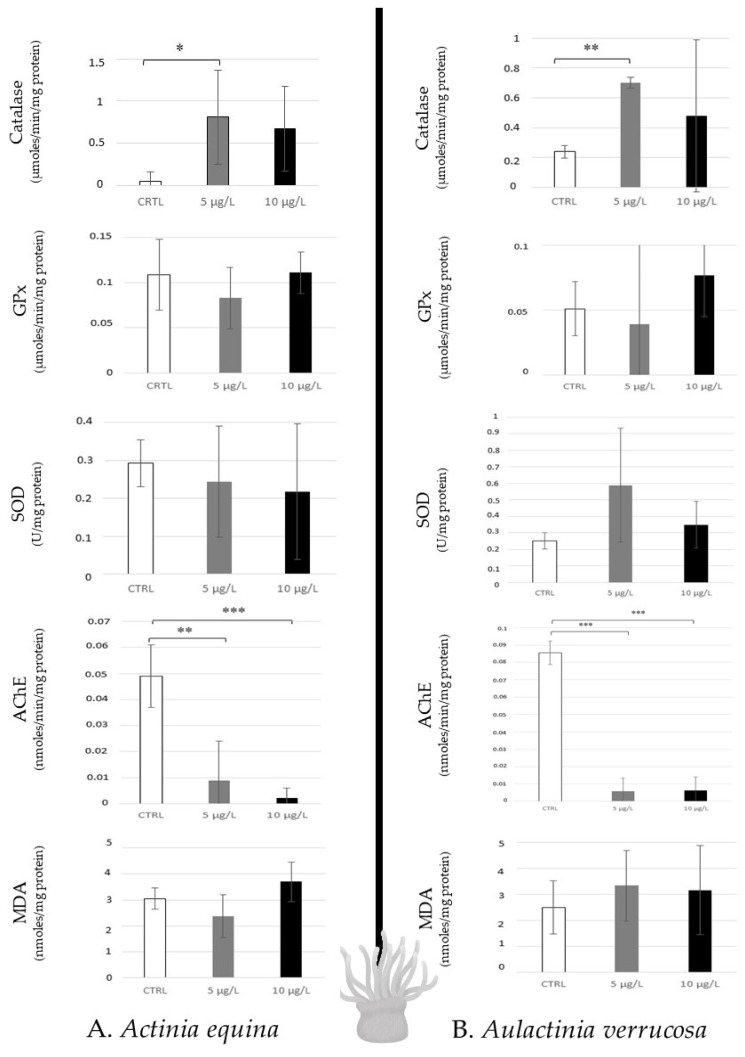
Antioxidative enzymatic responses (Catalase, GPx, SOD), acetylcholinesterase activity, and lipid peroxidation in sea anemones (*Actinia equina*, (**A**); *Aulactinia verrusoca*, (**B**)) after 14 days of caffeine contamination (control, CTRL; 5 µg/L; 10 µg/L). Distinct symbols indicate *p*-values: * *p* < 0.05, ** *p* < 0.01, *** *p* < 0.005.

The two species of sea anemones (*Actinia equina* and *Aulactinia verrucosa*), which are predators, revealed similar response profiles when they were contaminated with caffeine. A low disturbance was observed because of any modulation of antioxidative responses, and lipid peroxidation was noted, except for a catalase increase to 5 µg/L in *A. equina*. However, high AChE inhibition of the two concentrations was noted in the two species (Figure 4).

**Figure 5:**
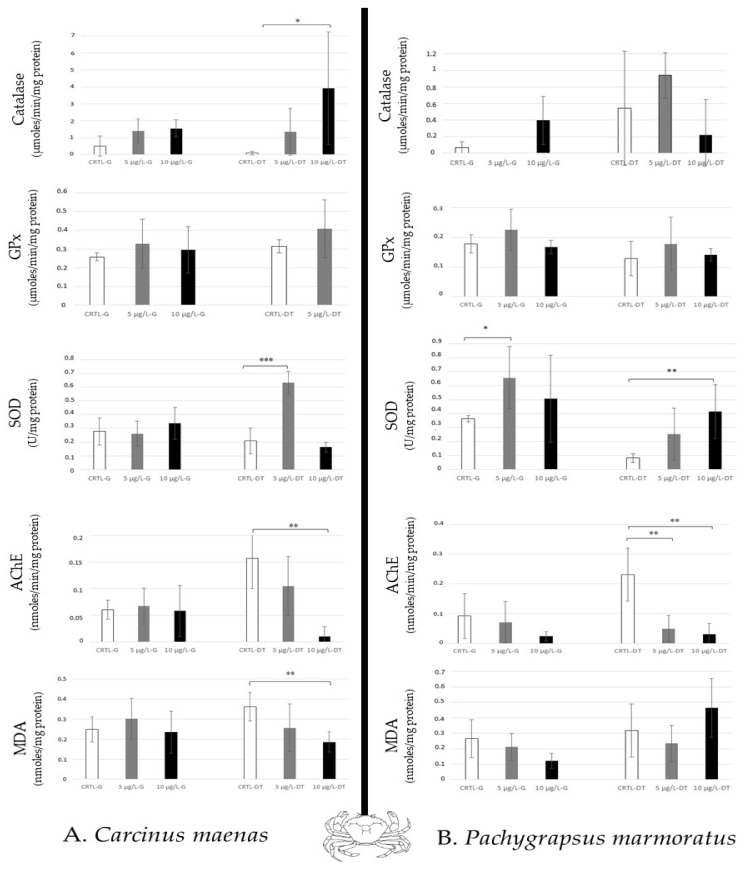
Antioxidative enzymatic responses (Catalase, GPx, SOD), acetylcholinesterase activity and lipid peroxidation in crabs (*Carcinus maenas*, (**A**); *Pachygrapsus marmoratus*, (**B**)) after 14 days of caffeine contamination (control, CTRL; 5 µg/L; 10 µg/L). The biomarkers were analysed in the gills (G) and the digestive tract (DT). Distinct symbols indicate *p*-value: * *p* < 0.05, ** *p* < 0.01, *** *p* < 0.005.

For the crabs (predator and scavenger) studied, *Carcinus maenas* (green crab) showed a high increase in SOD activity to 5 µg/L, and a significant increase in catalase to 10 µg/L in the digestive tract. Therefore, an AChE inhibition in the digestive tract was also observed at 10 µg/L (Figure 5A). *Pachygrapsus marmoratus* (marble crab) revealed a modulation of SOD activity in both tissues (to 5 µg/L in the gills, and to 10 µg/L in the digestive tract). High AchE inhibition was also noted in the digestive tract (Figure 5B). Thus, in crabs, a differential sensibility was noted between the two tissues (the digestive tract > the gills).

## 4. Discussion

Caffeine is currently detected in seawater, with hot spots of release in oceans along the coasts of Australia (11,000 ng/L), Brazil (1300 ng/L), China (3060 ng/L), Japan (8230 ng/L), and the USA (5860 ng/L), and in seas such as the Mediterranean (Italy: 1110 ng/L, Greece–Turkey: 3068 ng/L), and the Red Sea (7708 ng/L) [5]. Caffeine is now considered an omnipresent contaminant in aquatic ecosystems [7,8]. This compound is rapidly integrated into organisms directly by diffusion and via the trophic chain. Caffeine residues are effectively detected, for example, in algae [30], bivalves [31,32], coral reefs [33], and fishes [34]. The effects of caffeine noted in marine taxa are genotoxicity (crustaceans), oxidative stress (annelids, bivalves, crustaceans), lipid peroxidation (annelids, crustaceans), neurotoxicity (bivalves), mortality (annelids), reprotoxicity (crustaceans, echinoids), and development/growth inhibition (algae, crustaceans) [7,8].

In this study, we analysed the caffeine effects in several taxa occupying distinct ecological functions in marine ecosystems. Our choice was focused on filter (Bivalvia: *Magallana gigas*), grazer (Gastropoda: *Littorina littorea*), predator (Anthozoa: *Actinia equina*, *Aulactinia verrucosa*; and Malacostraca: *Carcinus maenas*, *Pachygrapsus marmoratus*) and scavenger (Malacostraca: *C. maenas*, *P. marmoratus*). This study is the first investigation of the impacts of caffeine on sea anemones and marine gastropods.

Caffeine exposure at environmental concentrations (5 µg/L (low), 10 µg/L (high)) after 14 days in laboratory conditions (salinity: 33 g/L, lighting: 15 L/9 N, 20 ± 1 °C, pH 7.4–7.5) induced significantly high inhibition of acetylcholinesterase (AChE) activity in six model species in both concentrations (Figure 3, Figure 4 and Figure 5). This observation confirms the neurotoxicity of caffeine in aquatic organisms that has been suggested in clams such as *Ruditapes philippinarum* after 14 days of exposure ranging from 5 to 50 µg/L [35], and in *Corbicula fluminea* (freshwater bivalve) at 5 µg/L [36]. A similar effect was noted in a marine amphipod (*Ampelisca brevicornis*) at 15 ng caffeine/g sediment (10 days) [37], and in freshwater fish (*Carassius auratus*) after 4 days of contamination (≥80 µg/L) [38]. The function of AChE is the hydrolysis of acetylcholine, which is a neurotransmitter in the neuronal system (central and peripheral neuromuscular parts). Thus, the neurotoxic effects of caffeine inhibiting AChE could highly disturb the neuronal system’s functioning [39].

The antioxidative responses generated by caffeine contaminations in species showed variable intensity, according to the taxa analysed. In molluscs, the oysters (filter–feeder) did not show modulation of the activity of the distinct enzymes tested (catalase, SOD, GPx) except for an increase in GPx in gills, at 5 µg/L, and no lipid peroxidation. This observation is surprising, because the bivalves have a high metabolism of filtration (2–5 L/hour), and we hypothesised that the *Magallana gigas* would show a high sensibility to caffeine accumulation. This absence of antioxidative responses and lipid destabilisation is in opposition to the results published on *Mytilus californianus* (mussels) which revealed induction of HSP70 in gills after caffeine exposure (0.05 and 0.2 µg/L for 10, 20 and 30 days) [7]. In *Mytilus galloprovincialis,* alteration of haemocyte parameters (total haemocyte count, haemocyte volume and diameter, proliferation) was noted after 14 or 21 days due to the combined effects of pH reduction (8.1 to 7.7 or 7.4) and caffeine exposure (0.05 and 0.5 µg/L) [40]. A dose-dependent reduction in haemocyte lysosomal membrane stability was also noted in *Ruditapes philippinarum* after 14 days of caffeine exposure (range: 1–50 µg/L) [7]. Therefore, [35] demonstrated DNA damage, an increase in lipid peroxidation and GST activity when clams (*Ruditapes philippinarum*) were exposed to caffeine (from 0.1 to 50 µg/L) after 14 days. More precisely, in the digestive gland of clams, detoxification metabolism, general stress, genotoxicity, neurotoxicity, and oxidative stress all increased after 14 days of exposure to 0.1 µg/L caffeine.

In opposition to oysters (bivalve filter), *Littorina littorea* (a gastropod grazer) revealed sensitivity to caffeine exposure, with high lipid peroxidation and activation of all antioxidative enzymes (catalase, GPx, SOD) tested in both concentrations (5 µg/L, 10 µg/L). This susceptibility of gastropods to caffeine has been indicated in the literature, because this compound is recognised as an effective repellent for slugs and terrestrial snails. Thus, some authors have proposed caffeine as an acceptable toxicant to control pests [41,42]. To explain the lipid peroxidation observed in periwinkle (grazer), which was not noted in oyster (filter), it is possible to hypothesise that these distinct molluscs have lipids with different structural properties.

Sea anemones (predator) were less influenced by caffeine exposure, because only catalase induction was noted at 5 µg/L in *Actinia equina*. In other conditions of contamination, few publications have mentioned that sea anemones show a low level of antioxidative enzyme activities against environmental stress (metals, oil, pesticides, etc.). For example, Ref. [43], estimating the SOD activity in *Exaiptasia pallida* contaminated by metals (Cu, Zn), concluded that this enzyme is an unsuitable biomarker for indicating stress in sea anemones. Ref. [44] also showed no regulation of gene expression of catalase and low down-regulation of SOD when *Nematostella vectensis* was exposed to polycyclic aromatic hydrocarbons [Benzo(a)Pyrene: 1 to 500 µg/L after 96 h]. Therefore, low modulation of catalase in *Aiptasia pallida* was mentioned after 24 days of metal exposure (Cd, Cu, Ni, Zn) [45]. Thus, it is possible that the analysis of the antioxidative responses in Sea anemones is not a good model for detecting the incidences of pollution. It is also possible to hypothesise that the sea anemone possesses a good detoxification mechanism which protects the animals against xenobiotics and contaminants. It could be interesting to characterise and study the gene expression and protein synthesis of the markers involved in biotransformation (P450, GST…) and efflux of pollutants (G protein…).

In the crabs, the digestive tract appeared more disturbed than the gills (Figure 5) showing lipid peroxidation, induction of catalase (only in *Carcinus maenas* at 10 µg/L caffeine after 14 days) and SOD activities, and inhibition of AChE. It was mentioned that *Carcinus maenas* (Portunidae) exposed to environmental concentrations of caffeine showed DNA damage, lipid peroxidation, EROD synthesis, and GPx expression in the hepatopancreas according to the caffeine concentrations tested (from 0.1 to 50 µg/L) compared to other tissues (gills, gonad, muscle) over 28 days of exposure [35]. Alteration of lysosomal membrane stability is also noted at 15 µg/L. Thus, this species is susceptible to caffeine. Thus, *Carcinus maenas* is considered a good marker for estimating environmental disturbance [19]. The caffeine also impaired reproduction in crustaceans, such as in *Ceriodaphnia dubia* (water flea, freshwater crustacean), and induced mortality after 48 h of exposure (LC_50_ = 60 mg/L) [46]. It was demonstrated that caffeine disturbs the hatching and the larval development of shrimps (*Palaemonetes pugio*) at 20 mg/L after 5 days [47], but the concentration used by the authors did not reflect the concentration measured in the marine environment.

Caffeine has also multiple effects on other marine organisms, such as in polychaetes for which catalase, GST, and CYP3A4 increase, and GSH/GSSG (reduced and oxidised glutathione) decreases were noted after 28 days of exposure at concentrations ranging from 9 to 18 µg/L) in *Diopatra neopolitana* and in *Hediste diversicolor* [48,49]. Upregulated HSP70 synthesis was also noted in coral–algal endosymbionts (*Symbiodinium* sp.) exposed to caffeine [50].

Thus, caffeine represents an environmental risk in the marine ecosystem, negatively affecting many taxonomic groups of animals occupying distinct ecological functions. This study completes the dataset analysed in the review published by [5], resolving our knowledge gaps about the effects of this compound on marine organisms. Thus, as suggested by [8], it seems to be necessary to define a targeted ecopharmacovigilance program on the caffeine in marine biota in the future.

In conclusion, it will be interesting to focus on the effects of caffeine on the neuronal system of marine invertebrates. Caffeine acts as an antagonist at adenosine receptors (A1 and A2a) in mammals and invertebrates (insects, nematodes, etc.). Caffeine consumption induces a cAMP increase through the inhibition of phosphodiesterases [51]. Therefore, it has been demonstrated in many invertebrates (crustaceans, insects, molluscs, sea urchins, etc.) that caffeine also interacts with ryanodine receptors, which increases the affinity of the receptors for Ca^2+^ [52,53,54,55]. Thus, the characterization of the ryanodine and adenosine receptors will allow us to study, by gene expression, the impact of caffeine on marine invertebrates. It could be interesting to select for this future investigation *Actinia equina* (cnidarian), *Carcinus maenas* (crustacean), and *Littorina littorea* (mollusc), which reacted well to caffeine exposure.

## 5. Conclusions

The production and human consumption of caffeine through foodstuffs (coffee, tea, drugs, etc.) worldwide induce a release of caffeine in aquatic ecosystems via wastewater. Thus, caffeine is an emergent pollutant in freshwater and seawater. This molecule induces multiple negative effects in fauna. In this study, we showed an increase in antioxidative responses (catalase, GPx, SOD) and lipid peroxidation, and high inhibition of acetylcholinesterase activity in all marine invertebrate models (bivalve, crabs, gastropod, and sea anemones). These results confirmed the neurotoxicity and oxidative effect of caffeine anteriorly noted in terrestrial (gastropods, insects) and other marine invertebrates (annelids, amphipods, clams).

## Figures and Tables

**Table 1:** Estimation of volume (cm^3^) of periwinkles (*L. littorea*), crabs (*P. marmoratus*), and oysters (*M. gigas*) used in this study, according to the formulas cited in the Material and Methods section.

Group	Species	Volume: Average ± SD (cm^3^)
Control	*Magallana gigas* (Bivalvia)	68.39 ± 16.95
5 µg/L		104.19 ± 27.26
10 µg/L		107.27 ± 21.72
Control	*Littorina littorea* (Gastropoda)	2.32 ± 0.67
5 µg/L		2.14 ± 0.57
10 µg/L		1.99 ± 0.40
Control	*Carcinus maenas* (Malacostraca)	64.03 ± 17.10
5 µg/L		62.35 ± 35.74
10 µg/L		56.37 ± 47.27
Control	*Pachygrapsus marmoratus* (Malacostraca)	9.12 ± 14.25
5 µg/L		6.11 ± 3.24
10 µg/L		5.65 ± 1.84

**Table 2:** Weight (g) of the sea anemone (*A. equina*, and *A. verucosa*) used in this study.

Group	Species	Weight: Average ± SD (cm^3^)
Control	*Actinia equina* (Anthozoa)	8.10 ± 4.98
5 µg/L		2.76 ± 1.27
10 µg/L		5.24 ± 1.86
Control	*Aulactinia verrucosa* (Anthozoa)	1.61 ± 1.37
5 µg/L		2.80 ± 0.59
10 µg/L		1.45 ± 1.09

## Data Availability

The data presented in this study are available on request from the corresponding author.
